# Metasurface Color Filters Using Aluminum and Lithium Niobate Configurations

**DOI:** 10.1186/s11671-020-03310-3

**Published:** 2020-04-09

**Authors:** Yu-Sheng Lin, Jie Dai, Zhuoyu Zeng, Bo-Ru Yang

**Affiliations:** State Key Laboratory of Optoelectronic Materials and Technologies, School of Electronics and Information Technology, Sun Yat-Sen University, Guangzhou, 510275 China

**Keywords:** Metasurface, Tunable color filter, Lithium niobite

## Abstract

Two designs of metasurface color filters (MCFs) using aluminum and lithium niobate (LN) configurations are proposed and numerically studied. They are denoted as tunable aluminum metasurface (TAM) and tunable LN metasurface (TLNM), respectively. The configurations of MCFs are composed of suspended metasurfaces above aluminum mirror layers to form a Fabry-Perot (F-P) resonator. The resonances of TAM and TLNM are red-shifted with tuning ranges of 100 nm and 111 nm, respectively, by changing the gap between the bottom mirror layer and top metasurface. Furthermore, the proposed devices exhibit perfect absorption with ultra-narrow bandwidth spanning the whole visible spectral range by composing the corresponding geometrical parameters. To increase the flexibility and applicability of proposed devices, TAM exhibits high sensitivity of 481.5 nm/RIU and TLNM exhibits high figure-of-merit (FOM) of 97.5 when the devices are exposed in surrounding environment with different refraction indexes. The adoption of LN-based metasurface can enhance FWHM and FOM values as 10-fold and 7-fold compared to those of Al-based metasurface, which greatly improves the optical performance and exhibits great potential in sensing applications. These proposed designs provide an effective approach for tunable high-efficiency color filters and sensors by using LN-based metamaterial.

## Introduction

Recently, the research progresses of metamaterials have advanced toward the realization of tunable metasurfaces that enables real-time control over their geometrical and optical properties, thus creating exceptional opportunities in the field of actively tunable metamaterials. They have been reported to span the visible [1–6], infrared (IR) [7–12], and terahertz (THz) [12–21] spectral ranges. As the unique optical properties in metasurfaces rely on the interaction between incident light and the nanostructure, desirable properties can be achieved by properly tailoring the shape, size, and composition of structure. Metasurfaces have enabled manipulation of near-field entities thereby allowing reconfiguration of intriguing features like magnetic response [1, 22], near-perfect absorption [14, 15, 23], transparency [17, 19], phase engineering [18, 20, 21, 24], MIR sensing and thermal imaging [10], resonance modulation [9] for many types of filters [1–5], and sensors [6–8, 12–14] applications.

To date, there are many active tuning mechanisms reported to improve the flexibility of metasurface. Most of designs are in the IR [10–12, 25–27] and THz [28–31] spectral ranges. Although there have been various reported approaches for actively tunable metasurfaces in the visible spectral range, such as mechanical stretching [32], electrostatic force [33], Mie resonance [34], liquid crystal [35], phase change material [36–38], and electro-optic material [39, 40] However, the number of studies on actively tunable metasurfaces in the visible spectral range is limited. Among the tuning mechanisms of electro-optical methods, graphene-based tunable metasurface recently draws a massive attention to researchers [41–43]. Besides, lithium niobate (LN) is one of the most important materials, which is regarded as the “silicon of photonics.” The approaches of metasurface on LN have drawn great attentions due to its wide transparency window, large second-order electro-optic coefficient up to 30 pm/V, and great compatibility with integrated photonics circuits [44]. Owing to its large second-order nonlinear susceptibility, the refraction index of LN can be tuned linearly by applying an electric field on it [44]. The incorporation of LN into the design of metasurface opens up the possibilities for ultrasensitive color filters with electro-optical active tunability. The above-mentioned active tuning methods are highly dependent on the nonlinear properties of natural material. They often lack desirable characteristics, such as large tuning range and uniform performance across the tuning range or requiring high drive voltage which severely limits their applications. Among these methods, actively tunable metamaterials using micro-electro-mechanical systems (MEMS) technology are widely studied due to the geometric characteristics of the metamaterial can be directly modified [26, 29]. MEMS-based tunable metamaterials often utilize a Fabry-Perot (F-P) cavity and then change the gap between two structural layers to tune the resonance [37, 45]. These structures can produce narrow absorption or transmission bandwidth with a large tuning range which makes it desirable for next generation applications.

In this study, two designs of metasurface color filters (MCFs) are presented. They are tunable Al-based metasurface (TAM) and tunable LN-based metasurface (TLNM) by using Lumerical Solution’s finite difference time domain (FDTD)-based simulations to investigate their optical characteristics in the visible spectral range. The propagation direction of incident light is set to be perpendicular to the *x*–*y* plane in the numerical simulations. The polarization angle of incident light is set as 0 and it means the electric vector oscillates along the *x*-axis direction as TM polarization. Periodic boundary conditions are also adopted in the *x* and *y* directions, and perfectly matched layer (PML) boundary conditions are assumed in both *z* directions. The reflection intensity is calculated by a monitor set above the device. The proposed devices exhibit active tunabilities and large tuning ranges. TAM and TLNM exhibit near-perfect ultra-narrowband absorptions spanning the whole visible spectral range. For the environmental sensing application, TAM exhibits high sensitivity while TLNM exhibits high FOM. These designs can be potentially used in high-resolution display, refraction index sensor and adaptive device in the visible spectral range.

## Designs and Methods

**Fig. 1:**
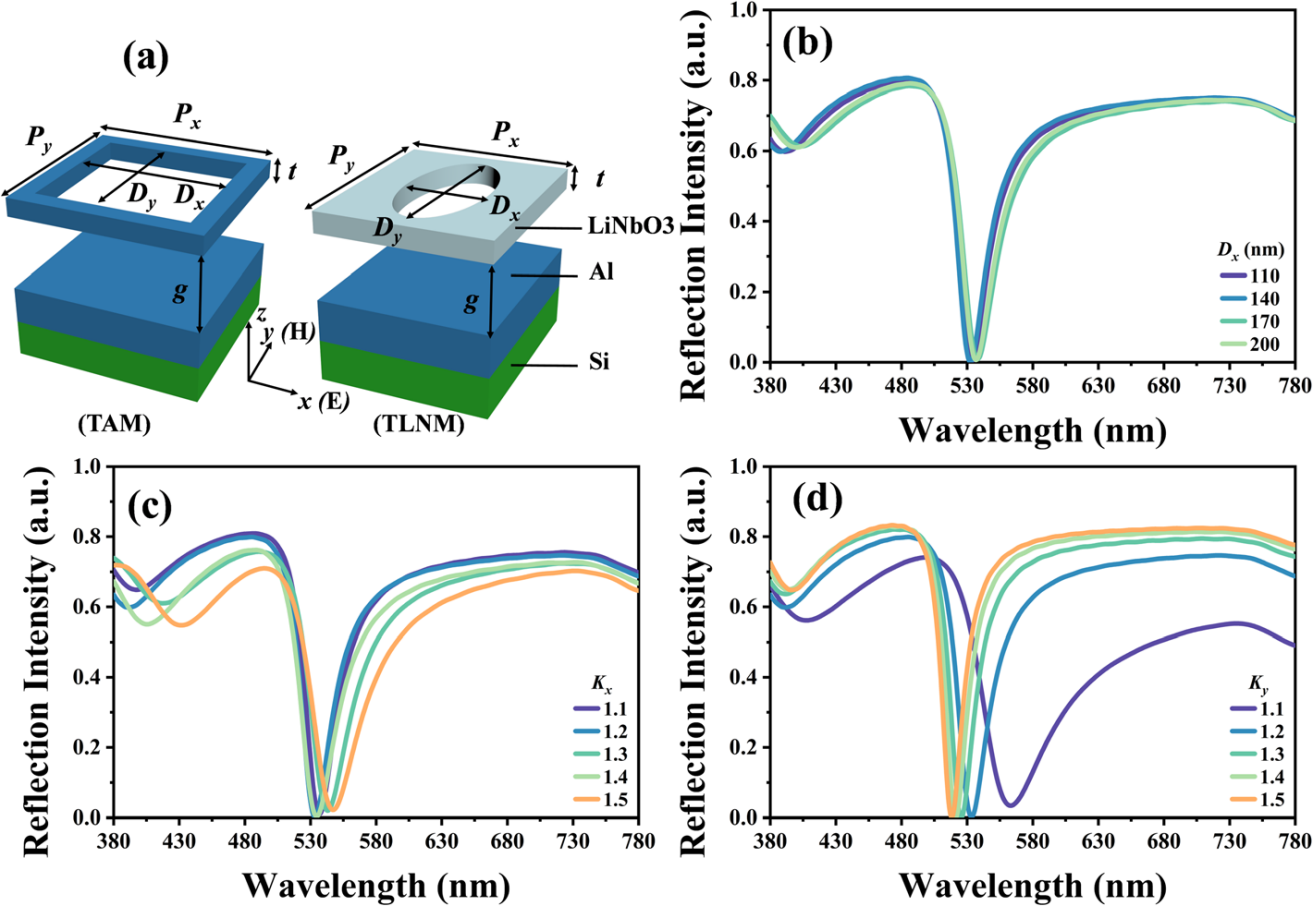
**a** Schematic drawings of TAM and TLNM. **b**–**d** The reflection spectra of TAM with different (**b**) *D*_*x*_, (**c**) *K*_*x*_, and (**d**) *K*_*y*_ values

Figure 1a shows the schematic drawings of proposed TAM and TLNM. They are composed of suspended rectangular Al and elliptical LN metasurfaces on Si substrate coated with an Al mirror layer atop. The gap between the bottom Al mirror layer and the top metasurface can be tuned by using MEMS technology to form a F-P cavity between these two layers. The corresponding geometrical dimensions are the length of the rectangular hole in Al metasurface and two axes of the elliptical hole in LN metasurface along *x*-direction (*D*_*x*_) and *y*-direction (*D*_*y*_), the periods along *x*-direction (*P*_*x*_) and *y*-direction (*P*_*y*_), the thickness of metasurface (*t*), and the gap between the metasurface and the bottom mirror layer (*g*). Here, we define the ratios of periods and the lengths of the rectangular Al metasurface and elliptical LN metasurface along *x*-direction and *y*-direction as *K*_*x*_ = *P*_*x*_/*D*_*x*_ and *K*_*y*_ = *P*_*y*_ / *D*_*y*_, respectively, to figure out the effective electromagnetic responses in the whole visible spectral range.

Figure 1b–d shows the reflection spectra of TAM by changing *D*_*x*_, *K*_*x*_, and *K*_*y*_ values, respectively. In Fig. 1b, the parameters are kept as constant as *D*_*y*_ = 200 nm, *g* = 450 nm, and *K*_*x*_ = *K*_*y*_ = 1.2. The near-perfect absorption spectra are maintained by changing *D*_*x*_ values from 110 nm to 200 nm. The resonance is at the wavelength of 535 nm. Figure 1c shows the reflection spectra of TAM with different *K*_*x*_ values. Other parameters are kept as constant as *D*_*x*_ = *D*_*y*_ = 200 nm, *g* = 450 nm and *K*_*y*_ = 1.2. The resonances are almost kept as constant in the wavelength range of 530 nm to 540 nm. Figure 1d shows the reflection spectra of TAM with different *K*_*y*_ values. The other parameters are kept as constant as *D*_*x*_ = *D*_*y*_ = 200 nm, *g* = 450 nm and *K*_*x*_ = 1.2. By changing *K*_*y*_ values from 1.1 to 1.5, the resonances are blue-shifted with a varying wavelength range of less than 60 nm. These results indicate that the impacts of *D*_*x*_, *K*_*x*_, and *K*_*y*_ values on the resonant wavelength of TAM are quite minor, which means that the proposed TAM possesses a high tolerance of manufacturing deviation for the variations of *D*_*x*_, *K*_*x*_, and *K*_*y*_ values. In the following discussions, *K*_*x*_ and *K*_*y*_ are kept as constant as 1.2 and *D*_*x*_ is set to be equal to *D*_*y*_ to investigate the active tunability of the proposed TAM and TLNM devices.

## Results and Discussions

**Fig. 2:**
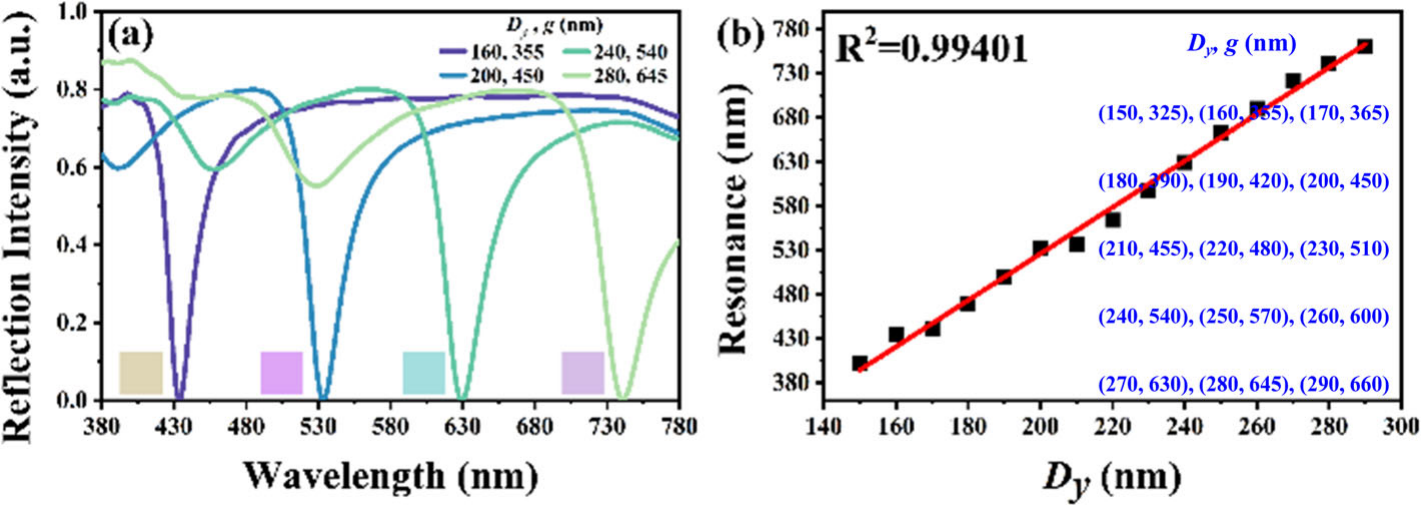
**a** Reflection spectra of TAM with different *D*_*y*_ and *g* values. **b** The relationship of resonances and *D*_*y*_ values

To increase the flexibility and applicability of proposed device, the metasurface is designed to be suspended so as to leave a gap between itself and the bottom mirror layer to form a F-P resonator and as a result of which, the incident light will be trapped in this gap and then absorbed by the device. Regarding to the *D*_*y*_ and *g* values are the main factors contributing to the shift of resonant wavelength, near-perfect absorption of TAM can be tuned in the whole visible spectral range by pairing *D*_*y*_ and g values as shown in Fig. 2a. Four pairs of *D*_*y*_ and *g* values are chosen to investigate the tunability of TAM. They are (*D*_*y*_, *g*) = (160 nm, 355 nm), (200 nm, 450 nm), (240 nm, 540 nm), (280 nm, 645 nm), respectively. By composing of *D*_*y*_ and *g* values, the perfect absorption can be realized at different wavelengths of 433.9 nm, 533.5 nm, 629.8 nm, and 740.9 nm. The inserted color images of Fig. 2a are the corresponding visible colors of reflection spectra to human eyes calculated by using CIE RGB matching functions to imitate the real colors on device surfaces. The relationship of resonances and *D*_*y*_ values is summarized and plotted in Fig. 2b. The resonances are red-shifted linearly spanning the whole visible spectral range by increasing *D*_*y*_ values from 150 nm to 290 nm. The corresponding correction coefficient is 0.99401. It shows a great tunability for the proposed TAM device. The resonant frequency of a F-P resonator can be determined by [46]

1 $$ {v}_q=\frac{qc}{2g} $$

where *q* is mode index, *g* is the length of F-P cavity, and *c = c*_*0*_*/n*, where *c*_*0*_ is the speed of light in vacuum and *n* is refraction index of the medium. This indicates that resonant frequency can be tuned by moving the suspended metasurface vertically in this proposed design, i.e., changing the *g* value.

**Fig. 3:**
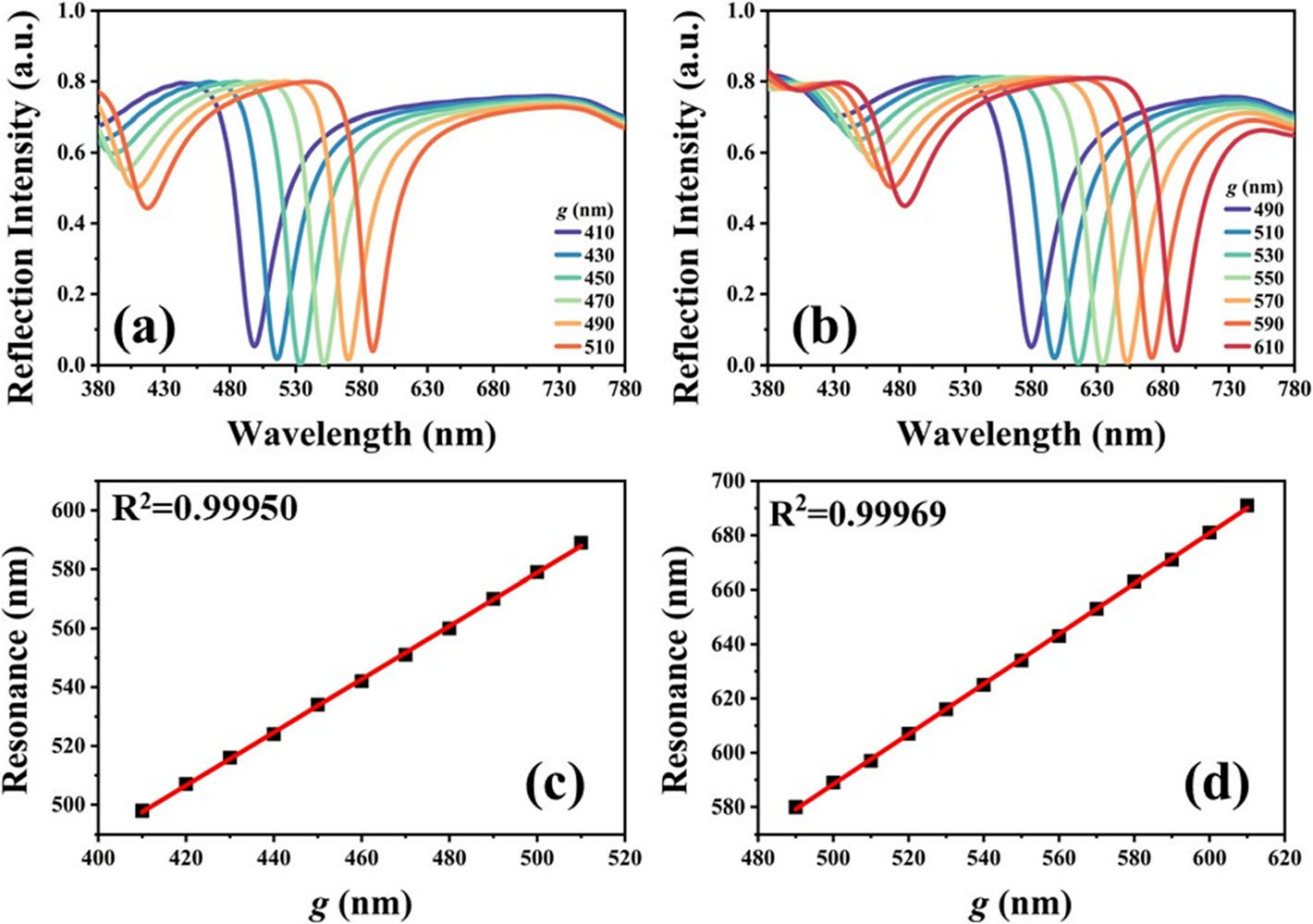
Reflection spectra of TAM with different *g* values under the conditions of **a***D*_*y*_ = 200 nm, **b***D*_*y*_ = 250 nm. **c, d** The relationships of resonances and *g* values of **a** and **b**, respectively

Figure 3 shows the reflection spectra of TAM with different *g* values under the conditions of *D*_*y*_ = 200 nm (Fig. 3a) and *D*_*y*_ = 250 nm (Fig. 3b), respectively. In Fig. 3a, the resonances are red-shifted from the wavelength of 490 nm to 590 nm by changing *g* values from 410 nm to 510 nm. The tuning range is 100 nm. The narrowest full width at half maximum (FWHM) of resonance is 29.9 nm for *g* = 470 nm. In Fig. 3b, the resonances are red-shifted from the wavelength of 580 nm to 691 nm by changing *g* values from 490 nm to 610 nm. The tuning range is 111 nm. The narrowest FWHM of resonance is 31.8 nm for *g* = 530 nm. The tuning range is 2-fold compared to that reported in the reference [39] and better than those reported in the references previously [37, 38, 40]. Figure 3c, d shows the corresponding relationships of resonances and *g* values of Fig. 3a, b, respectively. The resonances are red-shifted linearly by 9.2 nm per 10 nm increment of *g* value as shown in Fig. 3c, and by 9.0 nm per 10 nm increment of *g* value as shown in Fig. 3d. The tuning ranges are 90.5 nm and 110.7 nm, respectively. All reflection spectra are near-perfect absorptions. The corresponding correction coefficients are 0.99950 and 0.99969, respectively. Such designs of proposed TAM may serve as an ultrasensitive color filter or be used in various sensing applications.

**Fig. 4:**
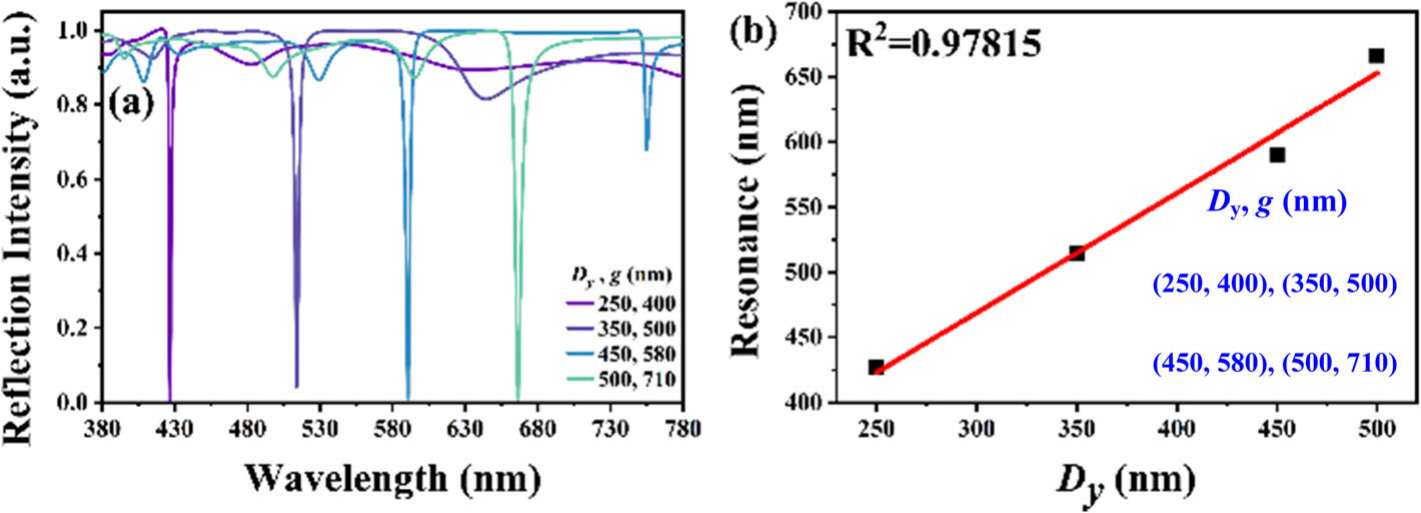
**a** Reflection spectra of TLNM with different *D*_*y*_ and *g* values. **b** The relationship of resonances and *D*_*y*_ values

To improve the performance of TAM in terms of FWHM and the tuning wavelength range while keeping the near-perfect absorption, TLNM is proposed and presented as shown in Fig. 1a. It is because the patterning of nanostructures always suffers the corner effect and fabrication deviation that the geometrical pattern is designed as elliptical hole. The parameters of *D*_*x*_ and *D*_*y*_ represent the lengths of macro-axis and minor-axis along *x-* and *y*-directions, respectively, while *K*_*x*_ and *K*_*y*_ parameters are kept as constant as 1.2 and *D*_*x*_ value is 110 nm. Figure 4a shows the reflection spectra of TLNM with four combinations of *D*_*y*_ and *g* values. *t* value is kept as constant as 200 nm. TLNM exhibits the characteristic of perfect absorption with an ultra-narrow bandwidth spanning the whole visible spectral range. The FWHM values of reflection spectra are 3 nm. Such ultra-narrow FWHM is contributed by the F-P resonance, which can be determined by

2 $$ \mathrm{FWHM}=\frac{\lambda_q^2}{2\pi g}\frac{1-R}{\sqrt{R}} $$

where *λ*_*q*_ is resonant wavelength, the subscript *q* is the mode index, *g* is the length of F-P cavity, and *R* is the reflectance of F-P resonator surfaces between bottom Al metasurface and Al/LN metasurface atop. FWHM value could be reduced as a result of higher reflection intensity of TLNM, which means the optical performance can be greatly improved by using LN material. The relationship of resonances and *D*_*y*_ values in Fig. 4a are summarized as shown in Fig. 4b. The resonances are red-shifted linearly spanning from 427 nm to 673 nm by increasing *D*_*y*_ values from 250 nm to 500 nm, and the corresponding correction coefficient is 0.97815. Hence, it demonstrates a linear tunability of the proposed device.

**Fig. 5:**
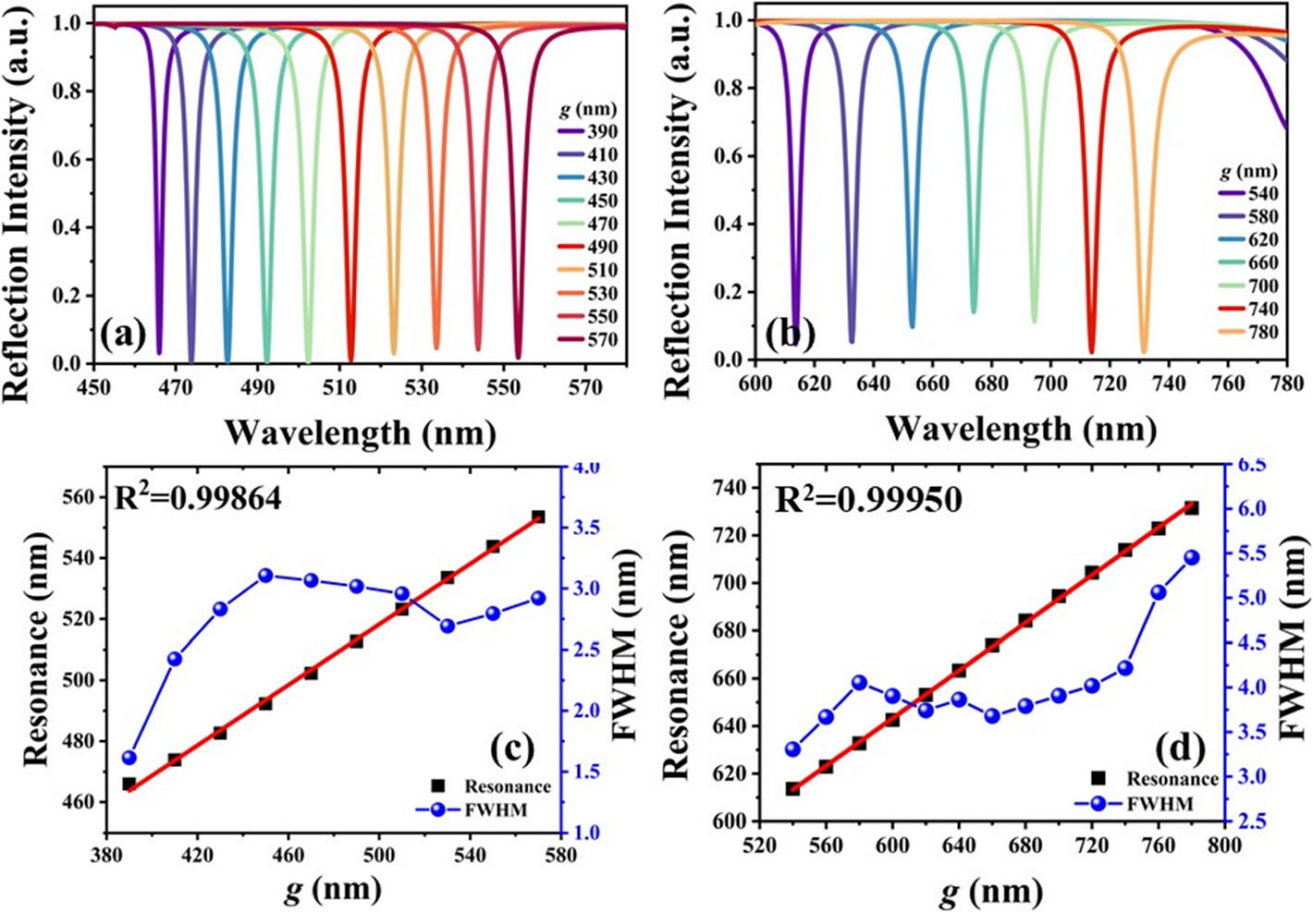
Reflection spectra of TLNM. Parameters are optimized for the maximum tunable range under the conditions of **a***D*_*y*_ = 350 nm, *t* = 210 nm, **b***D*_*y*_ = 450 nm, *t* = 280 nm. **c**, **d** The relationships of resonances, *g* values, and corresponding FWHM values of **a** and **b**, respectively

The suspended elliptical LN metasurface is moveable, which can be directly modified to achieve optical tunability by using MEMS technology. Figure 5a, b shows the reflection spectra of TLNM with different *g* values under two conditions of *D*_*y*_ = 350 nm, *t* = 210 nm, and *D*_*y*_ = 450 nm, *t* = 280 nm, respectively. In Fig. 5a, by increasing *g* values from 390 nm to 570 nm, the resonances are red-shifted from 465.9 nm to 553.5 nm. In Fig. 5b, by increasing *g* values from 540 nm to 780 nm, the resonances are red-shifted from 613.6 nm to 731.2 nm. Figure 5c, d shows the corresponding relationships of resonances, *g* values, and the corresponding FWHM values of Fig. 5a, b, respectively. The resonances are red-shifted quite linearly. The corresponding correction coefficients are 0.99864 and 0.99950 for two cases, respectively. For the case of *D*_*y*_ = 350 nm, *t* = 210 nm, the tuning range is 87.6 nm and the average FWHM value is 3 nm as shown in Fig. 5c. While for the case of *D*_*y*_ = 450 nm, *t* =280 nm, the tuning range is 117.6 nm and the average FWHM value is 4 nm as shown in Fig. 5d. It can be seen the narrowest FWHM value is 1.5 nm at the wavelength of 466 nm as shown in Fig. 5a and that is 3.2 nm at the wavelength of 615 nm as shown in Fig. 5b. They are compared with the results of proposed TAM designs, the FWHM values of TLNM are improved 10-fold at least keeping the perfect absorption. It is a great improvement of optical performance by using LN metasurface. These results indicate that TLNM can be potentially used in many applications such as ultrasensitive color filters, absorbers, detectors, and sensors according to these extraordinary characteristics of ultra-narrowband, perfect absorption, and large tuning range.

**Fig. 6:**
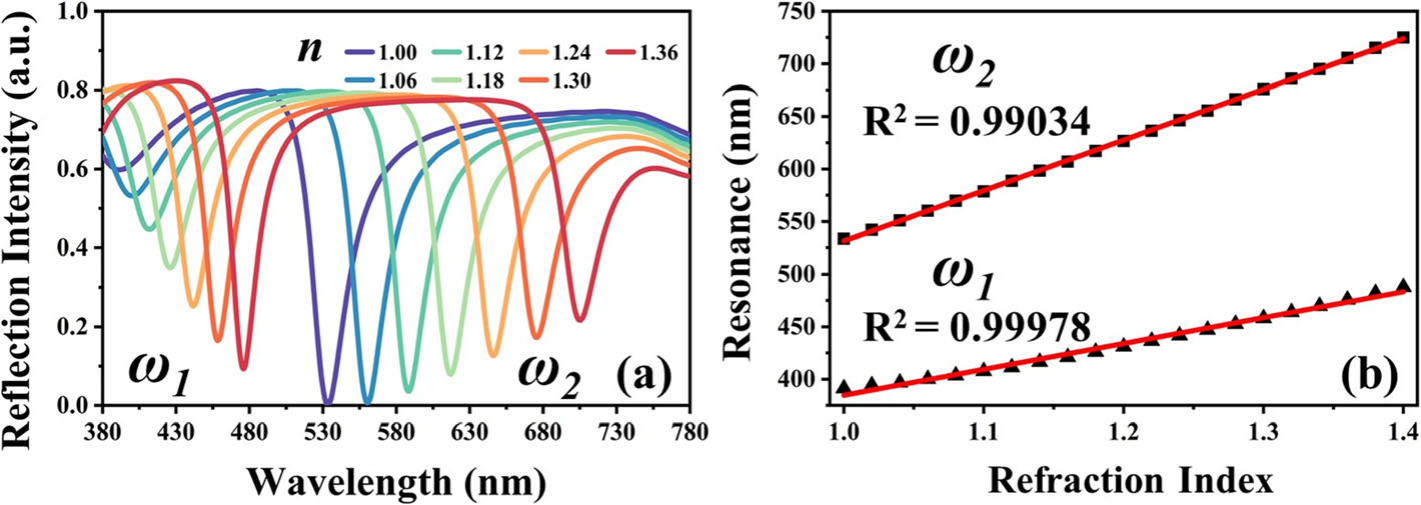
**a** Reflection spectra of TAM exposed in surrounding environment with different refraction indexes (*n*). **b** The relationship of resonances and *n* values

To further investigate whether TAM and TLNM devices can be implanted into practical applications, e.g., environment sensors, they are exposed in the surrounding environment with different ambient refraction indexes (*n*). Figure 6 shows the reflection spectra of TAM exposed in surrounding environment with different refraction indexes from 1.0 to 1.3. The geometrical dimensions of TAM are kept as constant as *D*_*x*_ = 110 nm, *D*_*y*_ = 200 nm, and *g* = 450 nm. There are two resonances red-shifted with tuning ranges of 84.6 nm (*ω*_*1*_) and 172.1 nm (*ω*_*2*_). The relationships of resonances and *n* values are summarized in Fig. 6b. The sensitivities are calculated as 246.7 nm/RIU and 481.5 nm/RIU, and the corresponding figure-of-merits (FOMs) are 11 and 14 for the first resonance (*ω*_*1*_) and second resonance (*ω*_*2*_), respectively. These higher sensitivities are caused from the narrow FWHM of resonances, which are 21.6 nm (*ω*_*1*_) and 34 nm (*ω*_*2*_). These characteristics are quite suitable for pragmatic sensing applications.

**Fig. 7:**
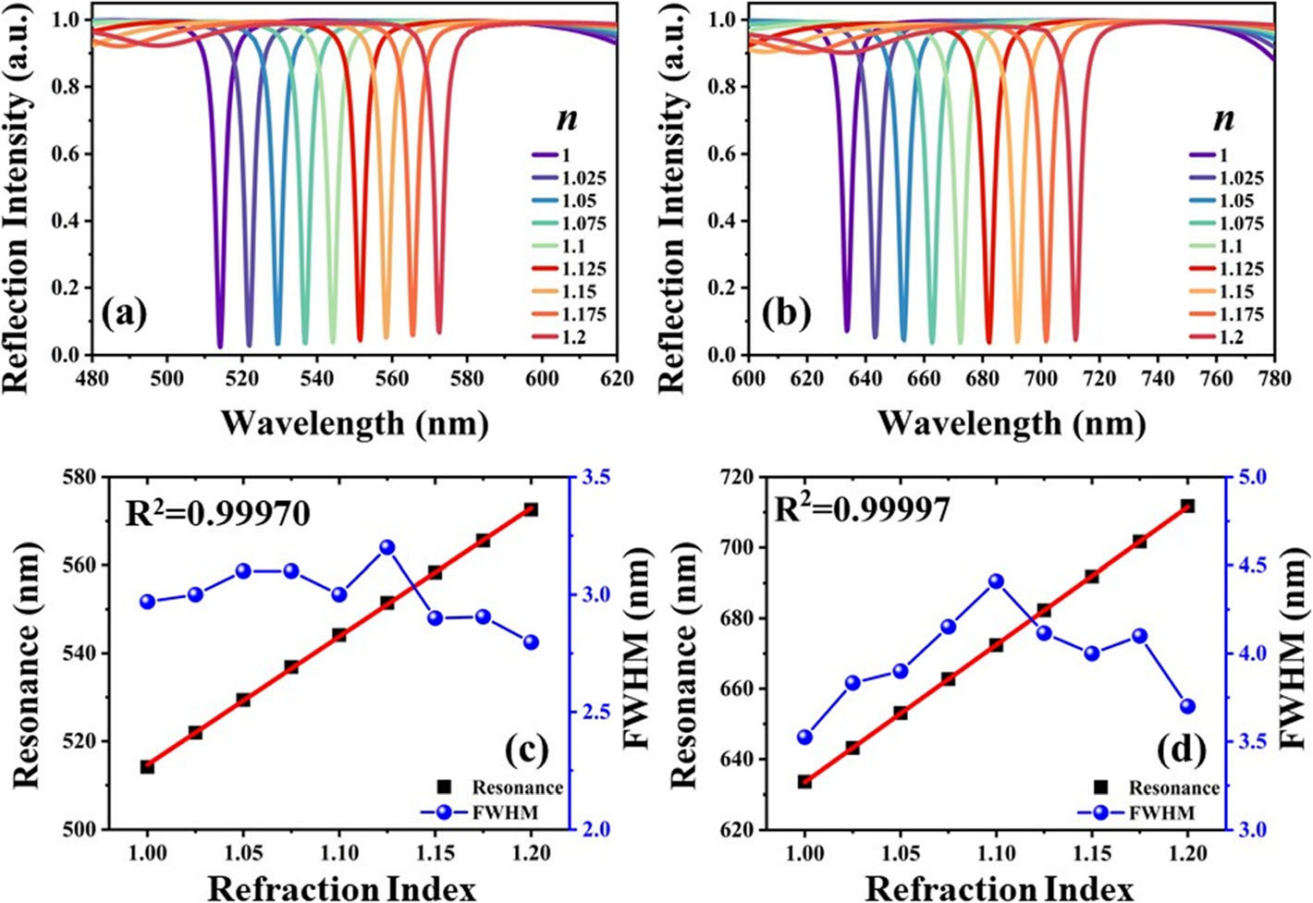
Reflection spectra of TLNM exposed in surrounding environment with different refraction indexes (*n*) under the conditions of **a***D*_*y*_ = 350 nm, *t* = 210 nm, *g* = 490 nm, **b***D*_*y*_ = 450 nm, *t* = 280 nm, *g* = 580 nm. **c**, **d** The relationships of resonances, *n* values and corresponding FWHM values, respectively

However, the drawback is that the reflection intensity of *ω*_*1*_ is relatively high and that of *ω*_*2*_ increases to greater than 20% as *n* increases to 1.3. To overcome this limitation, TLNM is designed to possess the stable optical properties owing to the characterizations of LN metasurface. Figure 7 shows the reflection spectra of TLNM exposed in surrounding environment with different *n* values under the conditions of *D*_*y*_ = 350 nm, *t* = 210 nm, *g* = 490 nm, and *D*_*y*_ = 450 nm, *t* = 280 nm, *g* = 580 nm as shown in Fig. 7a, b, respectively. In Fig. 7a, the resonances of TLNM with *D*_*y*_ = 350 nm, *t* = 210 nm, *g* = 490 nm are red-shifted with a tuning range of 58.4 nm by increasing *n* values from 1.0 to 1.2. While the resonances of TLNM under the conditions of *D*_*y*_ = 450 nm, *t* = 280 nm, *g* = 580 nm are red-shifted with a tuning range of 78.2 nm by increasing *n* values from 1.0 to 1.2. Within these two cases, TLNM exhibits near-perfect absorption, where the fluctuation of reflection intensity is less 5%. The reflection spectra are more stable than those of TAM. The relationships of resonances and *n* values are plotted in Fig. 7c, d for the two cases, respectively. For the condition of TLNM with *D*_*y*_ = 350 nm, *t* = 210 nm, *g* = 490 nm, the sensitivity and the average FWHM value are 291.4 nm/RIU and 3 nm, respectively. The corresponding FOM is calculated as 97 as shown in Fig. 7c. For the condition of TLNM with *D*_*y*_ = 450 nm, *t* = 280 nm, *g* = 580 nm, the sensitivity and the average FWHM value are 390.3 nm/RIU and 4 nm, respectively. The corresponding FOM is calculated as 97.5 as shown in Fig. 7d, which is enhanced 7-fold compared to that of TAM shown in Fig. 6. It means that TLNM shows better sensing performance to be used in the environmental sensor applications.

## Conclusion

In conclusion, we present two designs of tunable high-efficiency color filter based on suspended rectangular Al and elliptical LN metasurfaces on Si substrate coated with an Al mirror layer atop. By altering different compositions of *D*_*x*_, *g*, and *t* values of TAM and TLNM, the electromagnetic responses can perform perfect absorption with ultra-high efficiency spanning the whole visible spectral range. By increasing *g* values, the resonances of TAM and TLNM can be tuned 110.7 nm and 117.6 nm, respectively. For the environmental sensing application, TAM exhibits ultra-high sensitivity of 481.5 nm/RIU and TLNM exhibits ultra-high FOM value of 97.5. The FWHM of TLNM is enhanced 10-fold at maximum and FOM can be improved by 7-fold compared those of TAM. According to the above-mentioned characteristics of ultra-narrowband, especially FWHM of 3 nm for TLNM, perfect absorption and a large tuning range which are rarely reported in the visible spectrum simultaneously by implanting Al or LN metasurface, it indicates that proposed devices can be potentially used in many applications such as ultrasensitive color filters with high color purity, high resolution for display and imaging techniques, high-efficiency tunable absorbers desirable in integrated optics, refraction index sensors, etc. Among these applications, TLNM exhibits a performance with higher FOM and narrower FWHM while TAM possesses a higher sensitivity for refraction index sensors.

## Data Availability

All data generated or analyzed during this study are included in this published article.

## Abbreviations

MCFs: Metasurface color filters
LN: Lithium niobate
TAM: Tunable aluminum metasurface
TLNM: Tunable LN metasurface
F-P: Fabry-Perot
FOM: Figure-of-merit
IR: Infrared
THz: Terahertz
FDTD: Finite difference time domain
PML: Perfectly matched layer
